# ‘Disrespectful men, disrespectable women’: Men’s perceptions on heterosexual relationships and premarital sex in a Sri Lankan Free Trade Zone - a qualitative interview study

**DOI:** 10.1186/s12914-015-0040-4

**Published:** 2015-02-07

**Authors:** Malin Jordal, Kumudu Wijewardena, Ann Öhman, Birgitta Essén, Pia Olsson

**Affiliations:** Department of Women’s and Children’s Health, International Maternal and Child Health (IMCH), Akademiska sjukhuset, SE-751 85 Uppsala, Sweden; Department of Community Medicine, Sri Jayawardenapura University, Nugegoda, Sri Lanka; Umeå Centre for Gender Studies (UCGS), Epidemiology and Global Health, Umeå University, SE-901 87 Umeå, Sweden

**Keywords:** Masculinity, Free Trade Zones, Migrant women workers, Sexual and Reproductive Health and Rights, Qualitative, Sri Lanka

## Abstract

**Background:**

Gender norms have been challenged by unmarried rural women’s migration for employment to urban Sri Lankan Free Trade Zones (FTZ). Men are described as looking for sexual experiences among the women workers, who are then accused of engaging in premarital sex, something seen as taboo in this context. Increased sexual and reproductive health and rights (SRHR) risks for women workers are reported. To improve SRHR it is important to understand the existing gender ideals that shape these behaviours. This qualitative study explores men’s perspectives on gender relations in an urban Sri Lankan FTZ, with a focus on heterosexual relationships and premarital sex. Further, possible implications for SRHR of women workers in FTZs are discussed.

**Methods:**

Eighteen qualitative semi-structured interviews were conducted with men living or working in an urban Sri Lankan FTZ and were analysed using thematic analysis.

**Results:**

Two conflicting constructions of masculinity; the ‘disrespectful womaniser’ and the ‘respectful partner’, were discerned. The ‘disrespectful womaniser’ was perceived to be predominant and was considered immoral while the ‘respectful partner’ was considered to be less prevalent, but was seen as morally upright. The migrant women workers’ moral values upon arrival to the FTZ were perceived to deteriorate with time spent in the FTZ. Heterosexual relationships and premarital sex were seen as common, however, ideals of female respectability and secrecy around premarital sex were perceived to jeopardize contraceptive use and thus counteract SRHR.

**Conclusion:**

The ‘disrespectful’ masculinity revealed in the FTZ is reflective of the patriarchal Sri Lankan society that enables men’s entitlement and sexual domination over women. Deterioration of men’s economic power and increase of women’s economic and social independence may also be important aspects contributing to men’s antagonistic attitudes towards women. The promotion of negative attitudes towards women is normalized through masculine peer pressure. This and ambivalence towards women’s premarital sex are undermining the SRHR and well-being of women, but also men, in the FTZ. Awareness and counteraction of destructive gender power relations are essential for the improvement of the SRHR of women and men in the FTZ and the surrounding society.

## Background

This paper explores male perceptions of gender relations, with a focus on heterosexual relationships and premarital sex, in the context of an urban Sri Lankan Free Trade Zone (FTZ) factory area. Further, the paper discusses the possible implications of these perceptions for migrant women factory workers’ sexual and reproductive health and rights (SRHR).

In recent decades, the dominant ‘male breadwinner - female caretaker’ gender ideologies in Sri Lanka have been challenged due to the increased opportunities for women to migrate and participate in paid work within the country and abroad [1-4]. Masculinity in this context is described as drawing upon the nexus between status, respectability and acting ‘without harm’ [5]. A ‘real man’ has been described as being emotionally, physically and economically strong (breadwinners), able to protect women and their families and to achieve their goals [6]. De Silva [5] reiterates that men and young boys adopt different behaviours according to the context, whether in the presence of intimates and/or in confrontation with authority.

For women, the virtues of *lajja-baya* (shame-fear), characterised by shyness, naivety, docility, helplessness and chastity, and virginity at marriage are considered to be extremely important, and women’s failure to conform to these ideals often results in public ridicule [7]. Similar gender ideologies are prevalent in other contexts. Another example is the Latin American ‘machismo’ that represents an expression of masculinity that legitimizes dominance over other men and women. Its counterpart in that context, ‘marianismo’, implies women’s submissiveness, chastity, and self-sacrifice [8].

In the late 1970s, Sri Lanka opened up specific FTZs to enhance economic growth by facilitating the establishment of international companies, predominantly garment factories [9-11]. In response to this initiative, many young, unmarried women migrated from rural areas to work in the urban FTZs, and today around 80 percent of the employees are women. Earning money enables women to better tackle the changing realities characterized by rising poverty, unemployment among men [12], increased marital age [13], and a persisting demand for dowries [10]. The areas outside the FTZs, where most of the workers live, are characterised by an urban lifestyle with many shops and small hotels and restaurants, beauty parlours and cinemas, as well as exposure to the media; thus these areas are largely different from a village setting [10].

Men are said to be attracted to the FTZ areas to look for sexual experiences among the many unmarried women living there, while women workers in the FTZ are seen negatively for engaging in premarital sex [14]. Because premarital sex is not generally accepted in Sri Lanka, the SRHR of the unmarried woman are given little attention [15]. Cultural barriers to contraceptive use and restrictive abortion laws [16] contribute to unmarried women’s SRHR risks, although reliable data are lacking. Illegal abortion clinics in the FTZ areas cater to unmarried women’s desire for terminating pregnancies [10,14]. While some abortion providers do carry out medically safe abortions [17], others may not, and accessing these often results in health risks for these women [18].

Prevailing gender dynamics often diminish the agency of women and result in men being decision-makers in heterosexual premarital sex. To improve the SRHR of women and men, it is important to understand the existing constructions of masculinity that in turn shape sexual behaviour [19]. The dynamics and relational nature of gender construction and reproduction should be acknowledged when analysing men’s attitudes. Changes in social and economic contexts, as in the case of unmarried rural women’s migration to urban FTZs, affect male identity, men’s perception of women, and women’s and men’s sexual behaviour [20]. Therefore, contextualized knowledge of men’s understandings of masculinities and femininities is imperative for the development of theory, policies and interventions towards improved SRHR of women and men. While previous studies of Sri Lankan FTZ have investigated migrant women workers’ relationships, sexuality and SRHR risks [10,14,21], to our knowledge, there has been no investigation of men’s perceptions of these issues in this context.

The objectives of this study are to explore men’s perceptions of gender relations, with a focus on heterosexual relationships and premarital sex, in the context of a Sri Lankan FTZ. In the discussion section, we outline the possible implications for SRHR of migrant women workers in FTZs. The following research questions were explored: what are men’s views on constructions of masculinities and femininities in the FTZ?; how do they perceive the relationships between and within groups of men and women?; what are their perceptions of women’s and men’s involvement in premarital heterosexual relationships?; and what is their understanding of men’s responsibility for contraceptive use and SRHR risks?

## Method

### Design

As this topic is largely under-researched in this setting, we opted for an exploratory qualitative study design in order to grasp the complexities and nuances of the phenomena [22]. We conducted individual, semi-structured interviews and analysed the data using thematic analysis [23]. In reporting the findings in this study, we followed the RATS guidelines for reporting on qualitative studies.

### Theoretical framework

Gender relations and SRHR are the basic concepts employed in our analysis. The theoretical assumptions are that gender and gender relations are constantly changing and are products of social and cultural processes that vary over time and space. Power dynamics affect relational behaviours between and within individuals and groups of women and men [20,24]. Gender structures are most often representations of patriarchal systems giving men authority over women, even if not all men have the same amount of power [25]. According to Connell [24], different types of masculinities and femininities co-exist and masculinities are often acted out in opposition to femininity [26]. ‘Hegemonic masculinity’ implies an ideal of manhood in a given society, and demonstrating power over others is a key characteristic [24]. There are close links between masculinity and sexuality, and manifestations of power and violence.

The SRHR concept is important as it links health and human rights with sex and reproduction. SRHR embrace individuals’ and couples’ rights to a satisfying and safe sex life, the purpose of which is the enhancement of life and personal relations. Reproductive health concerns individuals’ capability and right to reproduce free of discrimination, coercion and violence [27]. The importance of men’s involvement in the promotion of SRHR has long since been universally agreed upon [27-29]. Yet, men’s perspectives are seldom taken into consideration in research on women’s SRHR [30].

### Recruitment and participants

An FTZ in the Western Province, close to the financial capital, Colombo, in Sri Lanka, was chosen for recruitment of participants to this study. The authors were acquainted with the area from previous work done with migrant women workers in the FTZ [21]. Purposive sampling was chosen to achieve a variety of perceptions. The criterion for participation was ‘being a man and working or living in the FTZ area’. To ensure sample variation, the first author, together with a research assistant, recruited participants from different sites and organisations located in the vicinity of the FTZ, including non-governmental organisations (NGOs) working with migrant women workers’ rights, shops, restaurants, enterprises, and factories. The research assistants were two male Sri Lankan social science students who were specially trained in recruitment, interviewing, transcription and the translation of data. To further maximise the variation of perceptions we recruited both married and unmarried men, as well as men working either inside or outside the factory area. Eighteen (18) men were approached and all agreed to participate following the obtaining of their informed consent.

The participating men all spoke Sinhala and were aged between 21 and 53 years. Five men originated from the FTZ area and the remainder had migrated to the FTZ from different towns and villages throughout the country. The men were between 21 and 53 years of age. The six participants aged 24 years and under were unmarried, and the remaining twelve who were aged 25 years or more were married (9), unmarried (2) and divorced (1).

### Data collection and handling

Semi-structured, individual interviews were conducted in 2009 by the first author and a research assistant. A thematic interview guide was used and the participants were asked to give their perceptions concerning: changes in a woman’s life after coming to the FTZ, relationships between women and men in the FTZ, the ideal man and woman, premarital sexual relationships in the FTZ, possible consequences of unprotected sex, and men’s and women’s knowledge regarding sexual and reproductive health. The interviews were held in privacy in English (4) or Sinhala (14) as per the participants’ preference. The interviews had a conversational style and the participants were encouraged to talk freely and reflect on the given topics [22]. Probing was done to clarify and expand on what had been said. After the interviews, the first author and the research assistant reflected on the content and the meaning of the perceptions of the participant. The interviews were tape-recorded, transcribed in Sinhala, translated into English by one research assistant and checked for accuracy by another. The interviews lasted 30 to 90 minutes. Data collection was stopped after 18 interviews as they gave rich and detailed information on the research question and it was agreed that the amount of data was sufficient for the planned analysis.

### Analysis

Thematic analysis [23] was employed as it is a systematic yet flexible method for identifying, analysing and reporting patterns of meaning within the data. We analysed the data inductively, looking for their underlying meanings. The analysis involved repeated readings of the entire dataset and identification of data extracts which are individually coded chunks of data related to the study aim. An example of the coding process is presented in Table 1. The codes were then collated and organised into themes which constituted meaningful patterns across the dataset. The process involved a reflexive back-and-forth movement between the entire dataset, data extracts and preliminary themes. Regular discussions within the multidisciplinary Swedish-Sri Lankan research team enabled a common understanding and provided grounds for the development of the themes. Finally, the themes were compared with the data extracts and with the entire dataset and minor adjustments were made. When themes representing patterns based on the latent meaning of the data had been formed (see Table 1), the significance and broader meanings were theorized and interpreted in relation to existing literature, as shown in the Discussion section.

**Table 1:** **Example of coding of interview text**

**Data extract**	Code*
‘There are those [men] like that [who take responsibility for contraceptives]. There are those who do not too. That is, those who are not planning a life here [in the FTZ] have no need for birth control. There is no need to work here and stay, no? Therefore there are men who feel that it does not matter if she gets into that situation [pregnancy] or not. They do what they want to and even relinquish the job here. They go off. They go to the village. There are those like that’.	Some men do take responsibility for contraceptive use while others do not; they do not care if women get pregnant and are likely to abandon a pregnant woman and the FTZ.

*This code was later part of the theme ‘Divergent masculinities - disrespectful womanizer or respectful partner?’.

### Ethical considerations

Ethical approval was provided by the Ethics Committee, Faculty of Medical Sciences at the University of Sri Jayewardenepura (approval number A 307). Permission to recruit participants inside the factory premises was obtained from the Board of Investment. The respective NGO authorities gave permission for recruitment at their sites. Before each interview, the participants were provided with oral and written information about the purpose and procedure of the study; of the measures taken to ensure confidentiality; and that participation was voluntary and could be ended at any time if the participants so wished. Then, after confirming they understood what their participation would involve, consent to participate in the study was given by each participant. Talking about sexuality is sensitive in Sri Lanka, therefore the interviewers probed with sensitivity to the participants’ possible discomfort. In reports, fictive names are used for participants and other persons, institutions and places to ensure confidentiality.

## Results

The interviewed men’s perceptions of gender relations in the FTZ, with focus on heterosexual romances and premarital sex, are presented in three themes, as seen below in Table 2. Quotes representing the major content are presented under each theme.

**Table 2:** **Themes on men's perceptions on gender relations with focus on heterosexual romances and premarital sex**

1.	Divergent masculinities - disrespectful womanizer or respectful partner?
2.	Migrant women workers’ moral degradation - from innocent to disrespectable
3.	Traditional ideals, modern realities – secrecy around sex jeopardizes prevention

1. **Divergent masculinities - disrespectful womanizer or respectful partner?**

Masculinity in the FTZ was expressed by the study participants as consisting of two dichotomised constructions of masculinity; the disrespectful womanizer and the respectful partner.

The disrespectful womanizer masculinity style was perceived as predominant and was acknowledged as being morally wrong by the interviewed men. It entailed an antagonistic and disrespectful attitude and behaviour towards women. Men resorting to antagonism were perceived as exploiting unmarried migrant workers, economically or sexually. Economic exploitation involved borrowing money or letting the FTZ women pay for the men’s food and housing. Sexual exploitation involved seeking outlets for their sexual desires by entering sexual relationships with unmarried women without truly loving them:

*“When you take boys, they connect with girls to fulfil their own needs. Only a few relate to girls in a good way. Those are the instances where girls are led to other things [sexual relations]”. (Saman, 27 years, unmarried)*

The disrespectful womanizers were described as those who trick unmarried women into sexual relationships with the promise of marriage, or who arrange fake marriages, as migrant women workers, most of whom were brought up in rural villages, were thought to value the cultural ideals of premarital virginity. Migrant women were seen as likely to aspire to having a secure financial future and upward mobility, resulting in perceptions of men pretending to be wealthier than they actually were, by borrowing expensive clothes and motorbikes. Men were predominantly seen to have a strong desire for sex and it was considered relatively easy to convince unmarried migrant women workers to engage in sexual intercourse. The disrespectfulness implied not only to seducing women into sex, but also to deliberately degrading women by making fun of them, spreading rumours, bragging and elaborating on sexual encounters with them, especially in all-men groups. A peer pressure to be seen to be dating as many women as possible was acknowledged. Furthermore, men’s fear of the consequences of failing to live up to this masculine image was depicted:

*“The competition among boys is to have as many girls as possible. One boy can be friendly with up to 3 or 4 girls. This is what boys speak to each other about. (…) Now, if there is a boy who has not been able to get friendly with a girl, the other boys tease him and make him ashamed”. (Sarath, 26 years, married)*

To be married elsewhere and have love affairs in the FTZ could be openly admitted within this masculinity style. Disrespectful talk and sexual and economic exploitation of women was believed to contribute to a degrading view of women in the FTZ, but it was also believed to be harmful for the men’s own mental state and their families’ reputation. Explanations for men’s reasons for engaging in disrespectful masculinity involved coming from a difficult family background, being unemployed, giving in to peer pressure, having financial difficulties as breadwinners in their families, and being involved in drinking and gambling. It was also seen to be related to men’s negative experiences with women in previous relationships, resulting in humiliation and wounded hearts:

*“Interviewer: Why do some men disrespect girls? Participant: Maybe his first love had not been faithful. She may have had another boyfriend while going out with him. Maybe he is trying to take revenge. (…) Maybe he met his first love after coming here [FTZ], when he was just eighteen or twenty. And if the girl happened to be a person who had gone through life, who had had many boyfriends, I mean if she had had relations with many boys, he might have been so genuine and finally heartbroken. That agony may push him to a state where he takes revenge from each and every girl in the society”. (Tharindu, 23 years, unmarried)*

The ‘respectful partner’ masculinity style was depicted as a morally upright masculine ideal by the study participants. This style involved a benign and responsible attitude and behaviour towards women, respecting, protecting and truly loving their partners and wives. Showing respect for a woman could involve not pushing a virgin female partner into sexual intercourse and thus jeopardizing her reputation. Consequently, responsible and respectful men could engage in non-penetrative sexual practices with their serious and steady virgin partners. Taking responsibility could also imply taking their partner to an illegal abortion clinic if having made her pregnant:

*“Sometimes taking responsibility means … now there are a lot of places [conducting illegal abortions] in this area where you can get rid of it [a pregnancy]. So normally they go to such places and get rid of the pregnancy”. (Saman, 27 years, unmarried)*

The interviewed men portrayed themselves as being committed in their romantic relationships and being respectful of women. Unmarried men denied having had premarital sex with their current partner; however, sex with previous partners, often married women who were assumed to be sexually experienced, was confessed. Despite admitting a peer pressure to engage in multiple romantic relationships simultaneously, the men claimed to be monogamous, and if unmarried, intending to marry their current partner even if they had not informed their parents about the relationships.

*“Interviewer: Do you have any plan to marry her? Can you please explain it? Participant: Definitely. That means I have to go to her home … Interviewer: Both your own and your girl’s parents agree with your affair? Or don’t your parents know? Participant: Still, they don’t know. Also our age is too low. We have to have a permanent job for ourselves. (…) We are both doing it secretly. We’ll tell them later. Interviewer: How long have you had this affair with her? Participant: Three years”. (Janaka, 21 years, unmarried)*

2. **Migrant women workers’ moral degradation - from innocent to disrespectable**

Migrant women workers were perceived to undergo a moral degradation in the FTZ; a gradient transition from innocent to disrespectable. Although FTZ factory work was seen as one of the few ways for unmarried women to improve a poor economic situation, it was believed that FTZ migration was more harmful than beneficial to them. In contrast to the traditional lifestyle in the village, the FTZ was seen as a modern society with negative influences.

The women’s young age, trustfulness, naivety, lack of experience with the FTZ environment, and competition to have a boyfriend, was perceived to make them vulnerable to disrespectful and dishonest men’s attempts to impress and seduce them. It was thus believed to be essential for the migrant women’s futures to have a profound understanding of the FTZ’s social environment. Such an understanding involved not trusting men blindly, ignoring their attempts to form relationships with them, selecting male partners wisely, and researching the man’s background before entering a love relationship. Older men in managerial positions portrayed themselves as father figures and protectors of migrant women workers, some talking about them as ‘innocent children’ in need of education about how to live in the FTZ society and avoid the risks involved in relating to men:

*“They are not adults, right? Therefore, it is, eh, one of our prime duties, as adults and as managers, to guide them in a proper manner, right? (…) And we, not only, and also we tell them, even walking in the road; ‘This is how you should walk. There would be boys, you know, coming around, coming behind you, maybe because you, that you are a pretty girl and all that. So, but, if you like to choose a boy, choose, do it, but be careful. Try to understand him first.’ So these are the things that we do, actually”. (Chamera, 50 years, married)*

With time, most migrant women workers were believed to change their attitude and behaviour due to exposure to the FTZ environment. They would lose their *lajja-baya* and start engaging in heterosexual love relationships. Furthermore, due to exposure to the media and a different lifestyle, they would change their mindset and way of dressing, consuming, and talking as a result of improved economic status, experience, knowledge, and self-confidence. They would also start interacting with each other and with men in new ways that deviated from what is perceived as normative for rural unmarried women:

*“If a girl is working in a garment factory we know that we have to be careful when we speak (laughs). As they know everything. Now, you understand what I mean, don’t you? When we as boys speak to them, we get to know. Nowadays, if going for a garment job, the girl would have moved much in society and have had many associations. (…) Anything can be discussed openly in their case. Now, take a girl who is at home [in the village]. Can we speak openly about any subject with them? They don’t speak, no, they say nothing. Now, when we speak to garment girls, sometimes they tell us their whole life story”. (Rukmal, 32 years, divorced)*

Migrant women workers’ acquired independence and self-confidence was somehow admired. However, the changes in manners that deviated from traditional notions of femininity and respectability were mostly perceived as negative. Further, it implied becoming less dependent on men, which could make women enter relationships with an unconcerned attitude towards their partners. These women were thought to not truly love their partners, but to care more about his economic and social status and thus could not be regarded as trustworthy. This attitude risked leaving financially unstable men being less attractive and vulnerable to being used by the women as temporary enjoyment:

*“I mean sometimes if a handsome guy, even an ordinary, good looking guy passes by, if there are two, three girls, and if they give him a look, he may stop for a while and may turn back to give his phone number and go. She calls him up. But she may already have a boyfriend. Sometimes, he may be in Army, Navy, or Air force and she just finds a partner from here, maybe a guy who works in a garment factory, to overcome her loneliness. But she, the girl will not be faithful to that guy. Because his job may not be good enough, his economic stability may not be that satisfactory. In such instances she just has a fake relationship”. (Tharindu, 23 years, unmarried)*

It was expressed that an ideal future wife would be a virgin. However, it was perceived possible to accept previous premarital sexual relations with other men if the woman was open about it from the beginning of the relationship. However, was she to omit such information until after marriage, this was believed to create problems with trust later on:

*“Now we get to know a girl, right? So we may also know this girl has fallen to the pits [have had sex] and is in trouble. But we have no problems with that. We know that and we are together with her. Now, if we get married and get to know afterwards, that is different. If we know that this girl has had relationships with others before, but now there is no one, only me. Then we can get married. Then we know the situation. We know her well. But if we get to know later on; then it is a problem”. (Rukmal, 32 years, divorced)*

3. **Traditional ideals, modern realities – secrecy around sex jeopardizes prevention**

There were diverging opinions about how common premarital sex was in the FTZ. While traditional ideals condemning premarital sex were still perceived as important, the social changes requiring unmarried migrant women workers to live without family surveillance were believed to make premarital sex a common phenomenon in the FTZ environment. Migrant women workers were seen as policing themselves as premarital sex was against cultural values and could imply severe social sanctions. Premarital sex was also perceived to happen only occasionally, to be a ‘slip’ not planned for and often taking place after the couple had been together for some time and was anticipating marriage. Love and sexual attraction were seen as strong forces, sometimes triggered by exposure to films and magazines with sexual content and were perceived as reasons for engaging in sex. Sex was perceived to be a natural part of human beings’ lives; however, due to society’s disapproval, it was believed that it was necessary for unmarried couples to keep their sexual relationships secret. Disagreement of society’s strong condemnation of premarital sex was expressed:

*“Now, I really cannot understand why you, why we, or the society, look at the entire thing [premarital sex] as a very dangerous or, or, or a thing that you should keep away from the general living”. (Chameera, 50 years, married)*

Innocence and trust, otherwise valuable qualities in unmarried women, was believed to make migrant women easily convinced to engage in premarital sex in a FTZ context. Premarital sex was perceived as risky for women. In the case of a premarital pregnancy, without the possibility to marry or have an abortion, women were believed to face social stigma and possibly exclusion from her family and community. Premarital pregnancies were usually depicted as a result of women’s voluntary involvement in premarital sex; thus the woman alone could be blamed for her desolate situation. However, if she had been raped, the woman was not seen as bearing the responsibility for a potential pregnancy. Moreover, rape was also described as a way of triggering women’s desire for sex:

*“Interviewer: If a girl refuses [sex], what will happen? Participant: Most of the time the boys force them. After that it [sex] is like an addiction. This is how it happens”. (Samantha, 26 years, unmarried)*

Men in the FTZ were perceived to have limited knowledge regarding safe sex. However, some awareness regarding ways to prevent unwanted pregnancies and sexually transmitted infections (STIs) was demonstrated. Perceptions regarding sexually active men’s willingness to take responsibility for using condoms varied. While often talking about heterosexual encounters in all-men groups, men were perceived to seldom talk about contraceptives or to articulate concerns regarding the consequences of unprotected sex such as pregnancies or STIs:

*“Interviewer: Do boys talk a lot about sexual relationships? Do they then also talk about birth control methods? Participant: No, they don’t talk. What do you mean? Interviewer: If they talk about sex and also about birth control? Participant: They don’t talk about birth control, but they talk about that other thing [sex]”. (Wickrama, 25 years, married)*

Sexually active migrant women workers were believed to be knowledgeable about safe sex, to take responsibility for protecting themselves against unwanted pregnancies and STIs and to be confident to ask their partners to use condoms or purchase and use oral contraceptives. However, it was also acknowledged that men would quickly assume women to be promiscuous if she was to negotiate contraceptive use with their sexual partner. Furthermore, purchasing contraceptives was perceived to be difficult for unmarried women as they ran the risk of being ridiculed or excluded if they were identified to be doing so:

*“I don’t think that the majority of women, unless they are married, will ever go to a shop [to buy contraceptives]. I don’t think so, they will not … Because once you go to a shop and buy it, if the people know that you are not married, then the problems start. Then she will be looked at as “Ok, this woman is not married and she still buys it. She is up to certain things”. (Chandara, 53 years, married)*

Knowledge regarding contraceptives was perceived as important in avoiding premarital pregnancies and STIs. At the same time, it was thought to be difficult for either women or men to take responsibility for contraceptive use when feelings of desire were in charge, even if they had sufficient knowledge:

*“Interviewer: Say that there are couples who have sex before marriage? Do they use birth control? Do you know of such? Participant: As I said earlier it depends on each situation … Meaning it depends on their mindset. If the girl trusts the boy a lot … considering the feelings of both the boy and girl it will depend if they decide at the time to use such [contraceptives]. According to their needs it will happen. I think they may not use it [contraceptives]”. (Wickrama, 25 years, married)*

## Discussion

This qualitative interview study with men in a Sri Lankan FTZ revealed two constructions of masculinity; the ‘disrespectful womaniser’ and the ‘respectful partner’. The former was considered predominant and immoral and the latter, morally upright. The migrant women workers’ moral values and ‘good’ behaviour upon arrival to the FTZ was perceived to deteriorate with time spent in the FTZ according to the participants. Strong cultural ideals condemning women’s premarital sex promoted secrecy and counteracted safe sex.

### Two forms of sexism

The masculinity constructions of the disrespectful womanizer and the respectful partner depicted in this study reflect the cultural discourses in Sri Lanka contrasting ‘good’ and ‘bad’ morals [7,11,31]. The disrespectful attitude towards women explicitly points towards men’s urge for exercising and demonstrating power over women and other men. The centrality of work in the construction of masculinity is an international phenomenon [32] reported also from India [33], Sri Lanka [3] Kenya and Tanzania [34]. A link between the ideals of manhood and the ability to exercise power and control over a space are reported by South Indian male textile factory workers. Their ability to achieve this ideal depended on the availability of resources, resulting in men with less resources feeling subordinated [33]. Men have conveyed a loss of self-respect and dignity in other Sri Lankan settings with high male unemployment combined with women’s increased work migration to the Middle East [3].

In the FTZ context, an increasing number of women have employment and money and occupy public space [10]. Women’s engagement in traditionally male dominated areas contest patriarchal dominance which might contribute to men’s real or feared social and economic marginalization and feelings of powerlessness. This could favour masculinity constructions that incorporate sexual exploitation of women as a way to demonstrate status, superiority and power over women and other men. Fear of marginalization within their male group could make it difficult for men to resist peer pressure to antagonistic behaviour towards women, despite perceiving it to be morally and personally wrong, as in the case of the men interviewed in the present study. However, the disrespectful masculinity in the FTZ cannot solely be due to some men’s experiences of unemployment and marginalisation. Such attitudes could not be possible without existing cultural-historical notions of masculinity that are associated with accepting and enabling men’s entitlement to sexually exploit women.

The respectful masculinity style, occupying the high moral ground and advocating the benevolent protection of women workers in the FTZ, as revealed in the present study, stands in contrast to the disrespectful masculinity style. A similar duality in masculinity is described by Glick and Fiske [35]. They discuss two forms of patriarchal sexism; hostile, and benevolent, implying a ‘virgin-whore’ dichotomization of women, and this duality constructs women as being doubly inferior to men. While hostile sexism shares similar traits to the disrespectful womanizer style, they describe benevolent sexism as ‘a subjectively positive orientation of protection, idealization, and affection directed towards women’ [35:763]. Hostile sexism, then, is easy to recognize and criticize because it is characterised by overt ferocity towards women, while benevolent sexism is less likely to be questioned because it is subjectively positive and characterizes some women as being pure and virtuous but weak creatures in need of and worthy of protection. Because women fall into either of these orientations, they are subjected to sexism on both levels. Benevolent sexism reinforces the importance of women adhering to certain ideals of femininity and disapproves of women who do not. Thereby, it contributes to the acceptance of men’s sexual exploitation of women who deviate from the ideals of femininity as they are not seen to be deserving of the protection of benevolent sexism. A similar way of reasoning was demonstrated among male university students in Sri Lanka, who justified the sexual harassment of women who they judged as failing to display *lajja-baya* [6]. The present study revealed the perception that migrant women workers undergo a moral degradation in the FTZ, from innocent to not respectable, as they become more knowledgeable and independent. Thus, it is probable that many migrant women workers will not be categorized as ‘pure, virtuous, and weak creatures’. Consequently, they are unlikely to experience the protection and respect that benevolent sexism could offer, and perhaps even be seen to be deserving of hostile sexism.

### Implications for migrant women’s sexual and reproductive health and rights

Over time, in the FTZs, unmarried migrant women workers were perceived to be having heterosexual relationships that often include sexual relations. Unmarried women’s virginity is strongly linked to whether or not they were regarded as respectable. Respectability is described by Skeggs [36] as a key factor for British low-income women’s position, and how women transgressing norms of female behaviour risk being regarded as disrespectable. In Sri Lanka, respectability and virginity is particularly important for unmarried women’s ability to remain included in her family and community [7,21,37]. In a South Asian context, female sexuality is perceived as both ‘voracious and in need for being kept in control and dormant, in need of being stimulated by a man to elicit the appropriate response’ [6,34]. When viewing women’s sexuality as insatiable, women who are not kept under control are assumed to engage in sexual behaviour, thus migrant women workers are easily labelled disrespectable. When men perceive women as disrespectable they are more likely to justify hostile sexism, which includes engaging in sexual relations with migrant women without due consideration of their SRHR, the possibility of getting married, inclusion in their community, or for their well-being. Previous studies have indicated that, to avoid being seen as disrespectable, pregnant unmarried women resort to illegal and unsafe abortions [16-18], involuntarily give up their child for adoption [37], or commit infanticide [38,39]. Furthermore, Marecek [40] reveals how women accused of sexual disrespectability may resort to acts of self-harm or suicide. Thus, being labelled as disrespectable in this context can have fatal consequences for both women and their children.

In the present study, sex was predominantly perceived as dangerous for unmarried women; however, it was also seen as natural and positive. The accepting attitude towards sexuality, regardless of marital status or gender, might be a generational shift and may imply an increased respect for women’s equal rights to sexual pleasure. However, liberalization of attitudes towards sexuality could also have negative consequences on women’s SRHR, as in the case of Ecuador, where more liberal views of premarital sexual activity placed further stress on young, unmarried women who were faced with the dilemma of both being chaste and sexually liberal [8]. Thus, when expectations of unmarried women to accept premarital sex co-exist with societal condemnation of sexually active unmarried women, their engagement in sex could increase. It may simultaneously constrain their search for SRHR information, negotiation of contraceptive use, or search for adequate and safe services if they become pregnant out of a fear of being perceived as promiscuous.

Non-penetrative sexual practices reduced the risk of pregnancies, although these practices were primarily employed to preserve the woman’s virginity. Sri Lankan men engaging in non-penetrative sexual practices out of concern for women’s virginity has been described by other researchers [6,41]. However, Ruwanpura [6] suggests that men also seem to opt for non-penetrative sexual practices out of fear of being forced to marry the woman if she becomes pregnant, as the Sri Lankan culture prescribes. Avoiding penetrative sex diminishes women’s negative health consequences. However, if men primarily avoid penetrative sex out of fear of being forced to marry, men residing temporarily in the FTZ area may be less motivated to engage in non-penetrative sex as they are difficult to track down in case of the woman becoming pregnant. This may leave FTZ migrant women workers especially vulnerable to reproductive health risks.

Men in the FTZ were perceived to be rather ignorant regarding sexual and reproductive health and sexually active women were expected to take responsibility for contraceptives. In contexts where the social acceptance for premarital sex is low, unmarried women face several barriers to obtaining and using contraceptives. Women trying to take responsibility for contraception are easily stigmatized as promiscuous and disrespectable, not only in Sri Lankan FTZs [14], but also, as reported, in Vietnam [42] and Thailand [43]. When men believe women take responsibility for contraceptives while in fact they do not, both genders then run SRHR risks.

While the present study did not particularly investigate sexual violence against women, rape was mentioned and trivialized in the interviews. Furthermore, rape was perceived to ‘trigger’ a woman’s desire for sex. In many contexts, saying ‘no’ in a sexual context is a marker associated with a submissive or feminine role [44]. Thus, a woman’s initial refusal to sex may be interpreted as ambiguous as it may be seen to comply with norms of *lajja baya*, thus encourages the man to persuade the women until she ‘agrees’. A recent study investigating violence against women in Sri Lanka, found that 20 percent of ever partnered men reported the perpetuation of sexual violence against an intimate partner, with the most common form being rape [45]. Rape has also been recognized as a problem in romantic relationships in the FTZs [14]. The finding that rape is not only trivialized, but also regarded as a way to ‘trigger’ a woman’s sexual appetite, is in line with the idea of female sexuality as ‘insatiable and in need of being stimulated by a man to elicit the appropriate response’ [6:34]. Nevertheless, it is a serious SRHR violation. Furthermore, we agree with Hewamanne [14] in that deliberately deceiving women into engaging in sexual intercourse without any considerations for the possible SRHR consequences for the women’s health and well-being, is also a form of violence against women.

Promoting gender awareness in FTZ factories could increase men’s responsibility for their sexuality and risk-taking behaviour. However, many of the partners of women factory workers do not work in the factories, and the promotion of gender awareness needs to be addressed on a societal level. Due to the high enrolment in schools in Sri Lanka [1,46], we suggest that the promotion of gender equality and sexual responsibility for men should be included in the school curriculum. Furthermore, promoting an understanding of men’s institutional power and violence in relation to women and the benefits for themselves of taking shared responsibility for women’s health and well-being would be an important step. Because men’s disrespectful behaviour and attitudes are often linked with abuse in childhood and social and economic marginalization [45], it is also important to place resources in the area of children’s security and well-being as well as promoting economic stability for both women and men. We advocate for the development of male-friendly clinics that encourage young men to reflect on the consequences of their own actions, allow them to express fears and hopes of intimacy, and help them to explore contraception in the context of pleasure and emotional intimacy. Not least, it is important to support women in taking control and responsibility over their own bodies and health.

### Methodological considerations

Conducting cross-cultural research always involves challenges and limitations [47]. In the present study, several measures were taken to ensure quality and trustworthiness. The composition of the research team with both Scandinavian and Sri Lankan members helped sharpen the methodology and interpretation of the data by providing both insiders’ and outsiders’ perspectives. The first and last authors spent month-long periods conducting fieldwork in the area over a four-year period to gain insights about the context. Working with research assistants required close collaboration but also gave opportunities to discuss interpretations of the interviews. Consistency in data collection and analyses was achieved through keeping the same principal investigators and the same overall study guide throughout the project. Repeated discussions among the authors regarding the interpretation of data as well as the cultural context facilitated a common ground of understanding and consensus on the meaning of the data. Descriptions of the study context and quotations from the interviews are provided to help the reader judge the interpretations and the transferability of the results to other settings.

Interviewing men from different geographical areas, socio-economic backgrounds, marital status, and ages, helped enable the understanding of the phenomena from different perspectives. We analysed these men’s perspectives as a unitary group, however it would be valuable to study variations between different groups of men based on socio-economic background, age and marital status, in future research, as this would provide a more nuanced and differentiated picture. Furthermore, because relations in all-men contexts are important in establishing masculinity, all-men-contexts become an arena where heterosexual orientation should be unambiguously displayed. It is therefore likely that, due to a social desirability bias, men portray themselves differently when being interviewed by a foreign, female researcher than they might in other contexts.

## Conclusion

The disrespectful masculinity revealed in the FTZ is reflective of the patriarchal Sri Lankan society norm that enables men’s entitlement and sexual domination over women. Deterioration of men’s economic power and increase of women’s economic and social independence may also be important aspects contributing to men’s antagonistic attitudes towards women. Fear of marginalization could hinder men’s resistance of peer pressure to antagonistic attitudes towards women, despite perceiving it to be morally and personally wrong. This and the ambivalence towards women’s premarital sex in the FTZ are undermining the SRHR and well-being of women, but also men, in the FTZ. Awareness and counteraction of destructive gender power mechanisms are essential for the improvement of the SRHR of women and men in the FTZ and its surrounding society.

## Abbreviations

FTZ: Free trade zone
SRHR: Sexual and reproductive health and rights
STI: Sexually transmitted infections
NGO: Non-Governmental Organisation
